# Smartphone Addiction and Sleep Disturbances Among Medical Students: A Cross-Sectional Study

**DOI:** 10.7759/cureus.91985

**Published:** 2025-09-10

**Authors:** Gayathri BH, Sravan JS, Sweta Shah, Sajidali S Saiyad

**Affiliations:** 1 Physiology, Pacific Medical College and Hospital, Udaipur, IND; 2 Forensic Medicine, Pacific Medical College and Hospital, Udaipur, IND; 3 Forensic Medicine and Toxicology, All India Institute of Medical Sciences, Bhopal, Bhopal, IND; 4 Physiology, Ram Krishna Dharmarth Foundation (RKDF) Medical College Hospital &amp; Research Centre, Bhopal, IND

**Keywords:** medical education, pittsburgh sleep quality index, sleep disturbances, sleep quality, smartphone addiction, smartphone addiction scale-short version (sas-sv)

## Abstract

Background

Smartphone use has become prevalent among young adults to the extent that it is affecting their sleep and overall well-being. First-year MBBS students, who are new to academic and environmental changes, may be particularly vulnerable to sleep disturbances. This study aims to explore the prevalence of smartphone addiction and sleep quality in this population.

Methods

This cross-sectional study was conducted among first-year MBBS students of a private medical college in central India. The study aimed to assess the prevalence of smartphone addiction and poor sleep quality, evaluate the correlation between them, and examine whether gender-wise differences were prevalent. Eighty-nine students voluntarily agreed to participate in the study. Data were collected using the Smartphone Addiction Scale-Short Version (SAS-SV) and the Pittsburgh Sleep Quality Index (PSQI). Qualitative variables were represented as frequencies and percentages; quantitative variables were reported as mean along with standard deviation. Chi-square was applied for statistical significance, and a p-value <0.05 was considered. Pearson correlation was used to analyze the relation between two variables.

Results

Among the 89 participants, 55.1% were found to have addiction to smartphones, and 53.9% had poor sleep quality. SAS-SV showed good reliability (α = 0.747), while PSQI showed lower internal consistency (α = 0.401). Significant associations were between components of PSQI, like subjective sleep quality, sleep efficiency, and total SAS-SV score. No significant correlation was found between total smartphone addiction scores and global sleep quality scores (r = 0.126, p = 0.254).

Conclusion

Smartphone addiction and poor sleep are very prevalent among first-year MBBS students. No significant association was found between poor sleep and mobile addiction, but PSQI components, subjective sleep quality, and sleep efficiency showed a significant association with mobile addiction. Awareness programs focusing on responsible smartphone use and healthy sleep habits are the need of the hour, especially in medical students, who are particularly vulnerable to academic stress.

## Introduction

Mobile phones have become a ubiquitous part of our daily lives. Although phones have made life more convenient and safer on one side, excessive use of mobile phones has led to numerous adverse side effects, such as tiredness, stress, headaches, and difficulty in concentration, which definitely affect academic performance [1]. The continuous use of something for the sake of relief, comfort, or stimulation, which often causes cravings when it is absent, is defined as addiction by the WHO [2]. Therefore, excessive mobile use can be considered a behavioral addiction.

Kwon et al. defined smartphone addiction as users underestimating the amount of time spent on their smartphones or being unable to regulate their use, resulting in negative consequences in daily life [3]. Research shows that 41.9% of Asian medical students had smartphone addiction, and it is positively correlated with poor sleep quality [4,5].

This may be due to rigorous academic schedules, prolonged study sessions, examination stress, and high parental expectations and digital media use [2]. Use of mobile phones, especially engaging in social media platforms, provides a sense of satisfaction and helps in escaping from real-world stress. This leads to a reinforcement mechanism, often causing increased mobile phone use [4].

Sleep is very important to maintain adequate physical and mental well-being. Lowry et al. reported that on average, 15% of college students were unsatisfied with their quality of sleep [6].

Many studies have reported that sleep disturbance will adversely affect cognitive skills, emotional intelligence, and academic performance [4,7]. This ultimately alters their work performance in the long run as medical practitioners, which can have a significant impact on healthcare.

This study aims to explore the prevalence and association of smartphone addiction and poor sleep quality among first-year MBBS students at a private medical college in Bhopal, a tier 2 city in central India.

## Materials and methods

Study design

This is a cross-sectional, observational study, conducted among consenting first-year medical graduates of a private medical college in Bhopal. In this study, we evaluated the relationship between smartphone addiction and poor sleep quality using standardized questionnaires.

Study population and sample size

**Figure 1:**
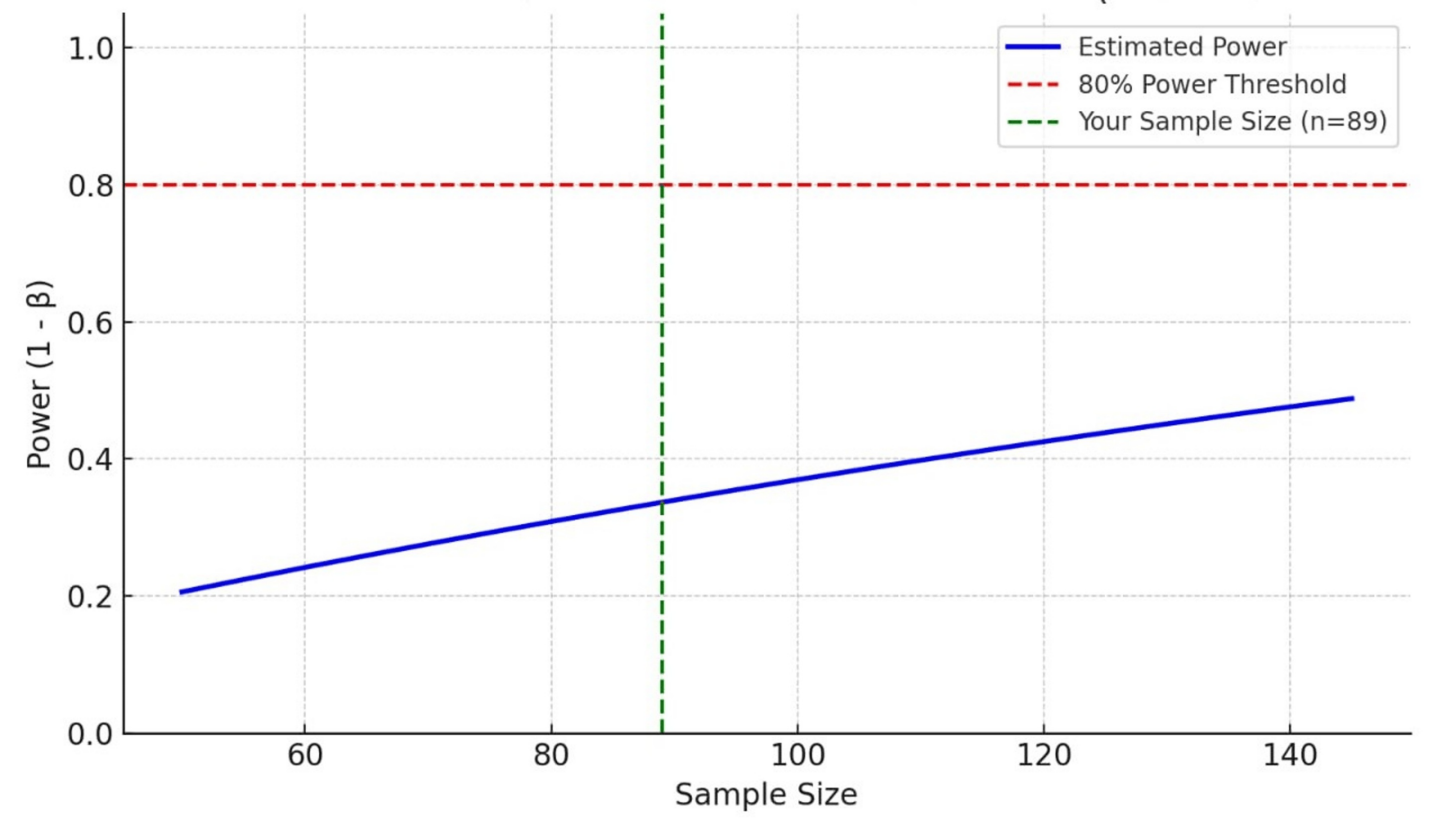
Post hoc power estimation for Pearson correlation (effect size = 0.3)

The total strength of the first-year batch of medical students was 150. A formal sample size calculation was not conducted prior to data collection due to the convenience-based nature of participant recruitment within the institutional setting. Out of 106 total consenting participants, 17 responses were excluded due to incomplete questionnaire responses. A total of 89 participants (n=89) voluntarily completed both questionnaires for this study. A post hoc power analysis was performed using G*Power software (version 3.1; Heinrich-Heine-Universität Düsseldorf, Düsseldorf, Germany) for Pearson’s correlation test (two-tailed). Assuming a medium effect size (r = 0.3), alpha = 0.05, and sample size = 89, the achieved power was approximately 0.85 (85%), indicating sufficient power to detect statistically significant correlations between Pittsburgh Sleep Quality Index (PSQI) and Smartphone Addiction Scale-Short Version (SAS-SV) scores as visually evident in Figure 1.

Inclusion and exclusion criteria

All first-year MBBS students who provided informed consent and completed both questionnaires were included in the study. Students with a diagnosed neurological disorder, as well as those with incomplete questionnaire responses, were excluded from the analysis. 

Data collection technique

Demographic data of all participants were recorded. They were briefed about the research objectives and were given instructions on how to fill out the questionnaires (Appendix A and B)**.** The questionnaires were administered online via Google Forms (Google LLC, Mountain View, CA, USA) with anonymized entries. Students completed them voluntarily at their own convenience, independent of examination schedules, and without supervision. They were assured of confidentiality and informed that participation would not affect academic grades; clarifications were provided on request.

Smartphone Addiction Scale-Short Version (SAS-SV)

SAS-SV (Appendix A) was used to assess the extent of mobile addiction [3]. SAS-SV is a self-reported measure for the assessment of smartphone addiction severity. The scale consists of 10 items, and responses were recorded by a 6-point Likert scale ranging from 1 (strongly disagree) to 6 (strongly agree). A total score of more than 31 for males and more than 33 for females in SAS-SV indicates smartphone addiction. This version, SAS-SV, was specifically designed for rapid screening in research settings and should not be confused with the original 33-item full SAS, which includes 6 factors.

Pittsburgh Sleep Quality Index (PSQI)

PSQI (Appendix B) was used to assess sleep quality [8]. PSQI is a seven-component questionnaire assessing subjective sleep quality, latency, duration, efficiency, disturbances, medication use, and daytime dysfunction. Global PSQI score was calculated from this, and those with a score >5 were classified as poor sleepers.

The SAS-SV questionnaire is licensed under Creative Commons (CC BY 4.0). The PSQI is copyrighted and reproduced with permission. The full English versions of the questionnaires are included in Appendix A and Appendix B, respectively, with proper citation and licensing.

Approval for the study was obtained from the Research Advisory Committee (RAC), the ethics committee of Ram Krishna Dharmarth Foundation (RKDF) Medical College Hospital & Research Centre, Bhopal, the medical university where the study was conducted, prior to initiation (Ref. No. RKDF/1350 dated 01/07/2023). The objectives of the study were (1) to assess the prevalence of sleep quality and smartphone addiction among first-year MBBS students, (2) to assess the correlation between smartphone addiction and sleep quality, and (3) to examine gender-wise distribution in smartphone addiction and sleep quality.

Statistical analysis

Data were entered into Microsoft Excel Office 365 (Microsoft Corp., Redmond, WA, USA) and analyzed using IBM SPSS Statistics for Windows, version 25 (IBM Corp., Armonk, NY, USA) [9]. Descriptive statistics such as frequency, percentage, mean, and standard deviation were used to summarize sociodemographic variables and questionnaire scores. Internal consistency of the SAS-SV and PSQI was assessed using Cronbach’s alpha. The association between categorical variables (e.g., gender and smartphone addiction, gender and sleep quality) was tested using the Chi-square test. A p-value <0.05 was considered statistically significant.

To examine the relationship between smartphone addiction and sleep quality, both Pearson correlation and the Chi-square test were used. Pearson correlation was applied to evaluate the association between continuous total scores of the SAS-SV and the global PSQI. Additionally, participants were categorized into groups (e.g., addicted/not addicted; good/poor sleep) based on standard cut-offs, and the Chi-square test was used to assess associations between these categorical variables.

## Results

Gender composition

**Figure 2:**
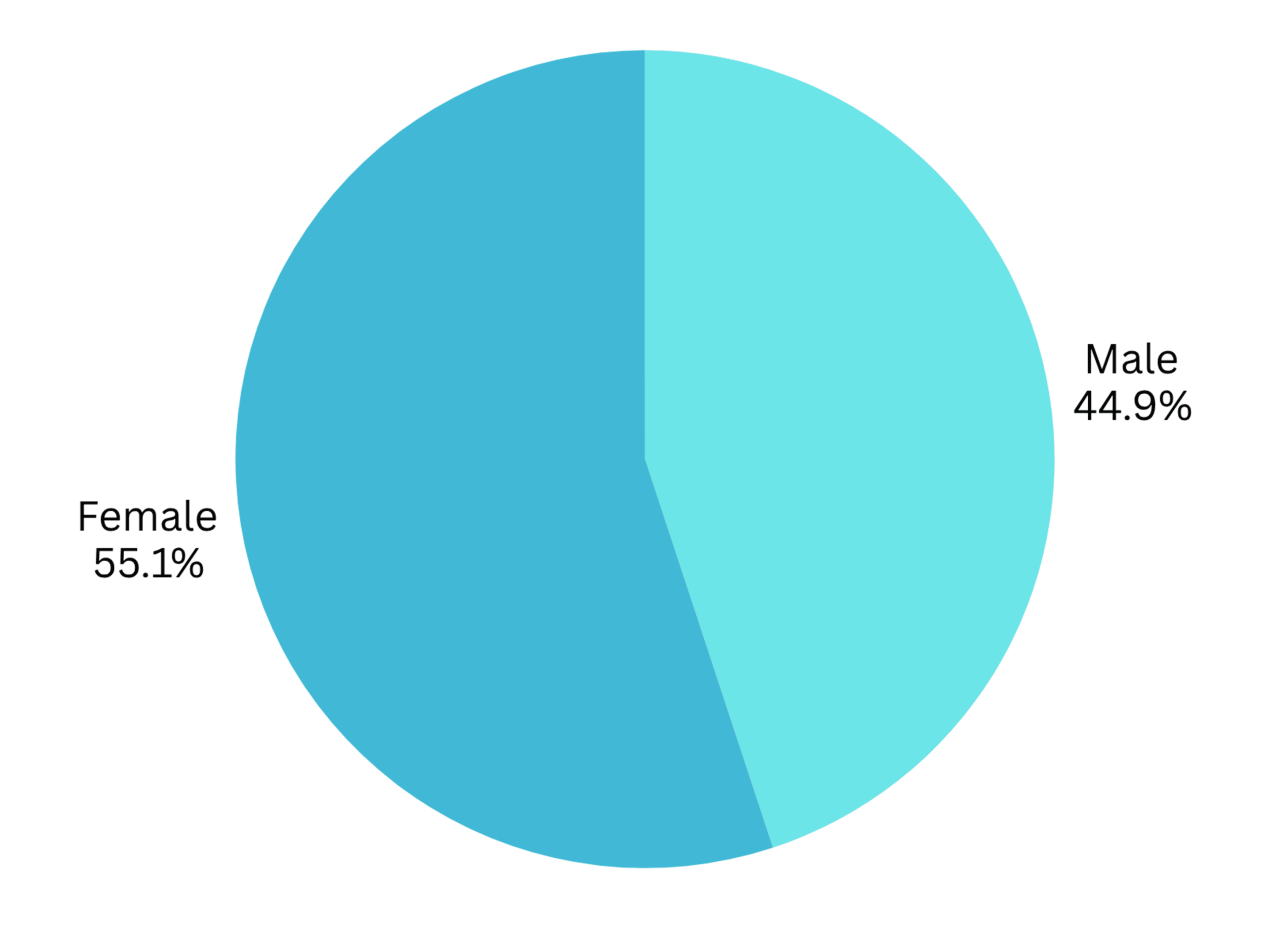
Gender distribution (n = 89)

Understanding the gender composition is essential for interpreting subgroup analysis, especially when examining variables like sleep behavior and smartphone usage patterns, which may vary between male and female medical students. Among the 89 participants, 40 (44.9%) were male and 49 (55.1%) were female medical students. The sample shows a slightly higher representation of female students compared to males (Figure 2). This near-balanced distribution provides a reasonable demographic spread for comparing gender-related differences in smartphone addiction and sleep quality.

Internal consistency of measurement scales

Cronbach’s alpha is a measure of internal consistency or reliability, indicating how well a set of items in a questionnaire measures a single underlying construct. Values can range between 0 to 1, with scores above 0.7 generally considered acceptable for reliability in social science research. The SAS-SV, comprising 10 items, demonstrated acceptable reliability with a Cronbach’s alpha of 0.747, indicating good internal consistency. In contrast, the PSQI, which contains 7 components, yielded a lower Cronbach’s alpha value of 0.401, suggesting relatively poor reliability in this sample. This could reflect contextual or cultural differences in the interpretation of sleep-related items or variability in responses, and highlights the need for cautious interpretation of PSQI results in this setting.

Prevalence of smartphone addiction among students

The distribution of smartphone addiction among the 89 first-year MBBS students based on the SAS-SV is shown in Table 1. Among the participants, 49 students (55.1%) were classified as addicted to smartphone use, while 40 students (44.9%) were categorized as not addicted. The classification was determined using gender-specific cut-off scores recommended for the SAS-SV (greater than 31 for males and greater than 33 for female students). The findings indicate that more than half of the students surveyed demonstrated signs of problematic smartphone use. This high prevalence highlights the growing concern regarding smartphone dependency in young adult populations, particularly among medical students who are already vulnerable to academic and psychological stress.

**Table 1:** Frequency of smartphone addiction

Addiction status	Frequency	Percentage
Addicted	49	55.1%
Not addicted	40	44.9%
Total (n)	89	100%

Patterns of smartphone use based on SAS-SV components

The SAS-SV, as reported by the students who participated in the study, is presented in Table 2. Among the components, the highest mean score was observed for the item “Using smartphone even when not necessary” (mean = 4.63 ± 1.54), indicating a strong compulsion among students to use their phones beyond intended necessity. This was followed by “Missing planned work due to smartphone usage” (mean = 4.34 ± 1.43) and “Constantly checking phone to not miss notifications” (mean = 3.79 ± 1.79), both suggesting frequent disruption of routine activities. Lower mean scores were noted for items such as “Having smartphone in mind even when not using it” (mean = 2.64 ± 1.90) and “Feeling impatient while not holding the phone” (mean = 2.94 ± 1.89), which reflect subtler psychological dependence.

**Table 2:** Mean and standard deviation of components of SAS-SV SAS-SV: Smartphone Addiction Scale-Short Version

SAS-SV component	Mean ± Standard Deviation
1. Missing planned work due to smartphone usage	4.34 ± 1.43
2. Won’t be able to stand not having smartphone	3.38 ± 1.70
3. Feeling impatient while not holding the phone	2.94 ± 1.89
4. Having smartphone in mind even when not using it	2.64 ± 1.90
5. Will never give up using my smartphone even when daily life is affected	2.91 ± 1.71
6. Constantly checking phone to not miss notifications	2.89 ± 1.61
7. Using smartphone longer than intended	3.07 ± 1.89
8. People around me say I use my phone too much	3.79 ± 1.79
9. Using smartphone even when not necessary	4.63 ± 1.54
10. The people around me tell that I am using my phone too much	3.13 ± 1.67

The distribution of smartphone addiction status among male and female first-year MBBS students based on the SAS-SV is explained in Table 3. Among males, 25 students (62.5%) were classified as addicted, while 15 students (37.5%) were not addicted. Similarly, among females, 32 students (65.3%) were found to be addicted, and 17 (34.7%) were not. Overall, 57 students (64.0%) met the criteria for smartphone addiction, while 32 (36.0%) did not.

**Table 3:** Association between gender with SAS-SV Values are presented as number (percentage). χ²: Chi-square test. No significant association was observed between gender and SAS-SV. SAS-SV: Smartphone Addiction Scale-Short Version

Gender	Addicted n (%)	Not addicted n (%)	Total n (%)	Test statistic	p-value
Male	25 (62.5%)	15 (37.5%)	40 (100.0%)	-	-
Female	32 (65.3%)	17 (34.7%)	49 (100.0%)	-	-
Total (n=89)	57 (64.0%)	32 (36.0%)	89 (100.0%)	χ² = 0.003	0.958

To assess the association between gender and smartphone addiction, a Chi-square test was performed. The result yielded a χ² value of 0.003 with a p-value of 0.958, indicating no statistically significant relationship between gender and addiction status (Table 3). This suggests that the prevalence of smartphone addiction was relatively similar across both genders in this sample. The lack of significance may reflect the widespread use and accessibility of smartphones among students, regardless of gender.

Descriptive analysis of Pittsburgh Sleep Quality Index (PSQI)

Detailed descriptive analysis of individual items from the Pittsburgh Sleep Quality Index (PSQI) revealed most common complaint was difficulty in maintaining sleep, with 36 participants (40.5%) reporting taking more than 30 minutes to fall asleep at least once a week. Seventeen (19.1%) students have experienced this three or more times a week. Night-time awakenings were also frequent; 31 participants (34.8%) reported waking up in the middle of the night or early morning at least once a week, and 15 participants (16.8%) had woken up three or more times a week.

Among the 89 participants, the average bedtime was 12:31 AM ± 1 hour 53 minutes, with the most common bedtime being 12:00 AM. The mean wake-up time was 7:12 AM ± 1 hour 16 minutes, with 8:00 AM being the most frequent (Table 4). The average sleep duration reported was 7.01 ± 1.45 hours, with 7 hours as the modal value. These findings indicate that while most students maintained a consistent wake-up time, there was greater variability in bedtimes, suggesting delayed sleep onset in a significant portion of participants, potentially affecting overall sleep quality.

**Table 4:** Summary of bedtime, wake-up time, and sleep duration among participants (n = 89)

Parameter	Mean ± SD	Mode
Bedtime	12:31 AM ± 1 hour 53 minutes	12:00 AM
Wake-up time	07:12 AM ± 1 hour 16 minutes	08:00 AM
Sleep duration (hours)	7.01 ± 1.45 hours	7

Regarding sleep disturbances, symptoms like bad dreams (34 participants, 38.2%) and nocturnal awakenings to use the bathroom (29 participants, 32.6%) were commonly reported. Pain-related sleep interruptions affected 29 participants (32.6%), of whom 10 (11.2%) experienced it frequently (three or more times a week).

The use of sleep medication was low, with only 9 participants (10.1%) reporting use at least once in the past month. However, daytime dysfunction was more prevalent; 42 participants (47.2%) reported trouble staying awake during daily activities at least once a week.

Subjective sleep quality ratings showed that 26% of participants rated their sleep as “very good”, 58% as “fairly good”, while 16% reported it as “fairly bad” or “very bad”. Components contributing most to poor sleep scores were Sleep Latency, Sleep Disturbances, and Daytime Dysfunction. Use of Sleep Medication showed minimal variability and was a negligible contributor to overall sleep quality scores as per responses from participants.

Sleep quality distribution

Among all participants, 46.1% (n = 41) were identified as good sleepers, while 53.9% (n = 48) were classified as poor sleepers (Table 5). The findings reveal a higher proportion of poor sleepers in the sample, indicating that sleep disturbances may be an important area of concern among medical students.

**Table 5:** Frequency of good and poor sleepers based on PSQI PSQI: Pittsburgh Sleep Quality Index

Sleep quality	Frequency (n)	Percentage (%)
Good sleep	41	46.1%
Poor sleep	48	53.9%
Total	89	100.0%

The mean and standard deviation were calculated (Table 6) for each of the seven components of the Pittsburgh Sleep Quality Index (PSQI) among the 89 first-year MBBS students. The highest mean score was observed for daytime dysfunction (1.60 ± 1.08), followed by sleep disturbance (1.30 ± 1.23) and sleep latency (1.21 ± 0.92), indicating that students commonly experienced issues in these domains. In contrast, components such as sleep efficiency (0.10 ± 0.48) and use of sleep medication (0.17 ± 0.53) had the lowest scores, suggesting these were not significant concerns within the sample. Subjective sleep quality (0.97 ± 0.75) and sleep duration (0.62 ± 0.87) showed moderate levels of disturbance.

**Table 6:** Mean of PSQI components PSQI: Pittsburgh Sleep Quality Index

PSQI component	Mean ± Standard Deviation
Subjective sleep quality	0.97 ± 0.75
Sleep latency	1.21 ± 0.92
Sleep duration	0.62 ± 0.87
Sleep efficiency	0.10 ± 0.48
Sleep disturbance	1.30 ± 1.23
Use of sleep medication	0.17 ± 0.53
Daytime dysfunction	1.60 ± 1.08

A Chi-square test of independence was performed to assess the association between gender and sleep quality, classified as good or poor based on the global PSQI (Table 7). The sample included 89 participants, comprising 40 male and 49 female students. Among males, 50.0% reported good sleep quality and 50.0% reported poor sleep quality. Among females, 42.9% had good sleep quality and 57.1% had poor sleep quality. Overall, 46.1% of participants had good sleep quality, while 53.9% had poor sleep quality. The chi-square test showed no statistically significant association between gender and sleep quality (χ² = 0.210, p = 0.646). These results suggest that sleep quality distribution is relatively similar between male and female participants in this sample. While a slightly higher proportion of females reported poor sleep compared to males, the difference was not significant.

**Table 7:** Association between gender and PSQI Values are presented as number (percentage). χ²: chi-square test. PSQI: Pittsburgh Sleep Quality Index

Gender	Good sleep n (%)	Poor sleep n (%)	Total n (%)	Test statistic	p-value
Male	20 (50.0%)	20 (50.0%)	40 (100.0%)	-	-
Female	21 (42.9%)	28 (57.1%)	49 (100.0%)	-	-
Total (n=89)	41 (46.1%)	48 (53.9%)	89 (100.0%)	χ² = 0.210	0.646

Correlation between overall smartphone addiction and sleep quality

A Pearson correlation analysis was conducted to examine the relationship between smartphone addiction and sleep quality using the total SAS-SV score and the Global PSQI score, respectively (Table 8). The results indicated a very weak positive correlation between SAS-SV and PSQI scores (r = 0.023, p = 0.829). This correlation was not statistically significant, suggesting that smartphone addiction levels, as measured by the SAS-SV, were not meaningfully associated with sleep quality in this sample.

**Table 8:** Pearson correlation between smartphone addiction (SAS-SV) and PSQI components r value = Pearson correlation coefficient; measures the strength and direction of a linear relationship between two variables. SAS-SV score has a significant correlation with sleep quality and sleep efficiency. SAS-SV: Smartphone Addiction Scale-Short Version; PSQUI: Pittsburgh Sleep Quality Index

Parameters	r value	p value
Sleep quality	0.226	0.03
Sleep latency	0.19	0.07
Sleep duration	-0.08	0.43
Sleep efficiency	0.22	0.04
Sleep disturbance	0.204	0.06
Sleep medication	0.05	0.59
Daytime dysfunction	0.1	0.2

The relationship between smartphone addiction, as measured by the SAS-SV, and various components of sleep quality assessed by the PSQI was examined using Pearson correlation analysis. Among the sleep parameters, significant positive correlations were observed between smartphone addiction and poorer sleep quality (r = 0.226, p = 0.03), suggesting that individuals with higher levels of smartphone addiction tend to report lower subjective sleep quality (Table 8). Additionally, a significant correlation was identified between smartphone addiction and sleep efficiency (r = 0.22, p = 0.04), indicating that greater smartphone use is associated with reduced sleep efficiency.

Other sleep dimensions, including sleep latency (r = 0.19, p = 0.07), sleep disturbance (r = 0.204, p = 0.06), sleep duration (r = -0.08, p = 0.43), sleep medication use (r = 0.05, p = 0.59), and daytime dysfunction (r = 0.1, p = 0.2), did not reach statistical significance (p > 0.05), though the positive directions of the correlations for several components suggest potential trends worth further investigation.

## Discussion

This study revealed a moderately high prevalence of smartphone addiction (n=49,55.1%) and a considerable prevalence of poor sleep quality (n=48,53.9%) among first-year MBBS students. Even though no significant correlation was found between overall smartphone addiction (SAS-SV) and global sleep quality (PSQI), specific components of PSQI, such as sleep quality (r = 0.226, p = 0.03) and sleep efficiency (r = 0.22, p = 0.04), showed statistically significant associations. This shows that while overall sleep quality may not always be impacted, certain domains of sleep are adversely affected by excessive smartphone usage.

Tettamanti et al. reported that overuse of smartphones was significantly related to increased sleep latency and reduced sleep quality in university students [10]. Many studies have pointed to increasing behavioral and psychological changes in people with smartphone addiction, emphasizing how constant blue light exposure can increase arousal and delay sleep onset [11].

Sleep disruption associated with smartphone use can be physiologically explained. Blue light emitted by screens interferes with melatonin secretion and disrupts the circadian rhythm [12]. These mechanisms explain why even short bursts of smartphone use prior to bedtime can reduce total sleep time and delay rapid eye movement (REM) cycles [13].

A longitudinal study by Lemola et al. reinforced these concerns by linking night-time media usage with long-term negative effects on academic performance and psychological well-being [14]. In a prospective study, reported that mobile phone use predicted subsequent stress, depression, and sleep disorders, especially among young adults [1,14].

The low Cronbach’s alpha (0.401) observed for PSQI in our study may indicate a limitation in the cultural or contextual adaptability of this scale among Indian students. It is important to note that all participants were undergraduate medical students, representing a homogenous academic stream. This lack of academic diversity may affect the generalizability of the findings, as sleep behaviors and interpretations of sleep-related questions might differ across disciplines. This necessitates further validation studies or potential localization of the tool for more reliable results in future research, and that too among various young adult populations.

In this study, we did not find significant gender differences in smartphone addiction and sleep disturbance. It is crucial to consider that all participants resided in on-campus hostel accommodations provided by the medical college. This shared residential and academic environment may minimize the influence of external variables such as family responsibilities, societal gender roles, or differing daily routines, which might otherwise contribute to behavioral differences. However, Roberts et al. noted that while patterns of usage differ by gender, addiction traits can manifest similarly in both [15]. Exelmans and Van den Bulck, in their research, indicated that bedtime mobile phone use, regardless of gender, is a strong predictor of shorter sleep duration and poor sleep quality [16].

Many studies across the world have highlighted moderate-to-strong associations between smartphone addiction and sleep disturbances [12]. Cha and Seo indicated that even middle-school children experience sleep loss due to rising smartphone addiction [17]. In our study, we could not establish an association between the global PSQI score and the total SAS-SV score, which may be due to low sample size. However, our study indicated that subjective sleep quality and sleep efficiency are poor in students with smartphone addiction. Twenge et al. showed that the strongest negative impact on sleep comes from portable screens like smartphones, as they are used close to bedtime and in bed [18].

Nurturing habits like setting the phone to do not disturb (DND) or airplane mode during bedtime can help reduce sleep disruptions. A systematic review suggested that more outdoor activities, psychological and cognitive behavioral therapy could be used to address depression, anxiety, and impulsivity associated with smartphone addiction [7].

There is a pressing need for multicenter studies with larger sample sizes involving medical students and students from other academic streams from different regions to improve generalizability. Also, there are scopes for future studies involving interventional trials testing the efficacy of mindfulness, digital hygiene programs, along with sleep, monitored in a sleep lab with polysomnography would give more objective results.

A limitation of this study is the relatively small sample size and homogenous study population, which may restrict the generalizability of the findings. In addition, the absence of objective sleep laboratory measurements limits the accuracy of sleep quality assessment. Future research with larger, more diverse populations and objective sleep measures is warranted.

## Conclusions

The study revealed a considerable burden of smartphone addiction and poor sleep quality among first-year MBBS students. Even though no significant correlation was found between overall smartphone addiction and global sleep quality scores, specific sleep components, namely (a) sleep quality and (b) sleep efficiency, showed a statistically significant association with smartphone addiction. These findings show that although smartphone overuse may not uniformly disrupt sleep, it does impact certain aspects of sleep health. This study underscores the importance of raising awareness about responsible smartphone use and importance of healthy sleep habits, especially in high-stress academic environments like medical colleges.

## Appendices

Appendix A 

Smartphone Addiction Scale-Short Version (SAS-SV) Questionnaire

**Table 9:** Smartphone Addiction Scale-Short Version (SAS-SV) Self-assessment of smartphone-related behaviors using a 6-point Likert scale (1 = Strongly Disagree to 6 = Strongly Agree). Source: Kwon et al. [3]. Permission to use the SAS-SV was obtained from the original publisher. This questionnaire is published under the Creative Commons Attribution License (CC BY 4.0), which permits unrestricted use, distribution, and reproduction in any medium, provided the original author and source are credited.

Item no.	Question	Response options (score)
1	Missing planned work due to smartphone usage	1 = Strongly disagree; 2 = Disagree; 3 = Weakly disagree; 4 = Weakly agree; 5 = Agree; 6 = Strongly agree
2	Hard time concentrating in class or while doing assignments due to phone use	Same as above
3	Feeling pain in wrist or back of neck while using smartphone	Same as above
4	Wouldn’t be able to stand not having smartphone with you	Same as above
5	Feeling impatient while not holding the phone	Same as above
6	Having smartphone in mind even while not using it	Same as above
7	Will never give up using my smartphone even when daily life is affected by it	Same as above
8	Constantly checking phone so as not to miss notifications	Same as above
9	Using my smartphone longer than I intended	Same as above
10	People around me say I am using my phone too much	Same as above

Appendix B 

Pittsburgh Sleep Quality Index (PSQI) Questionnaire

**Table 10:** Pittsburgh Sleep Quality Index (PSQI) Instructions: The following questions relate to your usual sleep habits during the past month only. Your answers should indicate the most accurate reply for the majority of days and nights in the past month. Please answer all questions. Source: Buysse et al. [8]. Permission to use the PSQI was obtained from the copyright holder.

Item no.	Question	Response options/frequency labels
1	Usual bedtime during the past month	Record time (hh:mm, AM/PM)
2	Usual time to fall asleep each night (minutes)	Record number of minutes
3	Usual wake-up time during the past month	Record time (hh:mm, AM/PM)
4	Hours of actual sleep per night (may differ from time in bed)	Record hours
5a	Trouble sleeping: Cannot get to sleep within 30 minutes	0 = Not during past month; 1 = <1/week; 2 = 1–2/week; 3 = ≥3/week
5b	Trouble sleeping: Wake up in the middle of the night or early morning	Same as above
5c	Trouble sleeping: Have to get up to use the bathroom	Same as above
5d	Trouble sleeping: Cannot breathe comfortably	Same as above
5e	Trouble sleeping: Cough or snore loudly	Same as above
5f	Trouble sleeping: Feel too cold	Same as above
5g	Trouble sleeping: Feel too hot	Same as above
5h	Trouble sleeping: Had bad dreams	Same as above
5i	Trouble sleeping: Have pain	Same as above
5j	Trouble sleeping: Other reason(s)	Describe reason; then same frequency coding as above
6	Overall sleep quality during the past month	0 = Very good; 1 = Fairly good; 2 = Fairly bad; 3 = Very bad
7	Frequency of taking medicine to help sleep (prescribed or OTC)	0 = Not during past month; 1 = <1/week; 2 = 1–2/week; 3 = ≥3/week
8	Trouble staying awake during driving, meals, or social activity	Same as above
9	Problem keeping up enthusiasm to get things done	0 = No problem; 1 = Slight problem; 2 = Somewhat of a problem; 3 = Very big problem
10a	(If bed partner/roommate) Loud snoring	0 = Not during past month; 1 = <1/week; 2 = 1–2/week; 3 = ≥3/week
10b	Long pauses between breaths while asleep	Same as above
10c	Legs twitching or jerking while you sleep	Same as above
10d	Episodes of disorientation or confusion during sleep	Same as above
10e	Other restlessness while you sleep	Describe reason; then same frequency coding as above
